# Serological, Genetic, and Biochemical Insights into Celiac Disease Diagnosis and Vitamin D Deficiency in Romanian Children: A Comprehensive Cohort Study

**DOI:** 10.3390/ijms26136251

**Published:** 2025-06-28

**Authors:** Luciana Alexandra Pavelescu, Ileana Delia Sabau, Gabriela Sanda-Dira, Alexandra Antonela Iacata, Antoanela Curici

**Affiliations:** 1 Department of Cellular and Molecular Biology and Histology, “Carol Davila” University of Medicine and Pharmacy, 050474 Bucharest, Romania; luciana.pavelescu@umfcd.ro; 2 Medical Genetics Department, Carol Davila University of Medicine and Pharmacy, 050474 Bucharest, Romania; ileana-delia.sabau@drd.umfcd.ro; 3 Synevo, 060201 Bucharest, Romania

**Keywords:** anti-DGP, anti-tTG, celiac disease, vitamin D, EMA

## Abstract

A large cohort of Romanian children suspected of celiac disease (CD) received comprehensive evaluation through this study regarding serological, genetic, and biochemical markers. This study investigated the relationships between anti-tissue transglutaminase (anti-tTG), anti-endomysium antibodies (EMAs), anti-gliadin deamidated (DGP) antibodies, and HLA genotyping. A strong association was observed between high anti-tTG IgA titers (>100 U/mL) and EMA IgA positivity, with a 95% concordance rate. Furthermore, anti-tTG IgA positive correlated with a significant prevalence of DGP antibodies, suggesting the complementary diagnostic role of DGP antibodies in equivocal cases. Genetic testing for HLA-DQ2/DQ8 alleles validated their association with celiac disease susceptibility, with 50% of the studied patients exhibiting these markers. The research reveals that vitamin D insufficiency affects a large number of children with anti-tTG antibodies, thus requiring both screening and supplementation practices. Furthermore, associations with other autoimmune conditions were explored, including thyroid and diabetes-related autoantibodies. This research demonstrates why CD diagnosis and management require a complete approach that combines serological tests with genetic evaluation and prompt intervention for related health conditions.

## 1. Introduction

Celiac disease (CD) is a chronic immune-mediated enteropathy that is induced by oral consumption of gluten in genetically predisposed individuals, particularly those carrying HLA-DQ2 or HLA-DQ8 alleles [1]. Epidemiological data suggest that CD is increasingly prevalent, with a global incidence estimated at around 1.4%, and some studies reporting even higher rates, increasing by approximately 8.1% annually between 1950 and 2010 [2]. Serological markers have grown in significance for both diagnosis and patient follow-up, even though intestinal biopsy, which shows villous atrophy and increased intraepithelial lymphocytes, remains the gold standard for diagnosis. Anti-tissue transglutaminase (anti-tTG) IgA, anti-endomysium antibodies (EMAs) IgA, and anti-gliadin deamidated (DGP) antibodies IgA and IgG are currently the three antibody tests that are frequently employed in clinical practice. So, CD-specific antibodies are regarded as highly specific diagnostic tools, particularly in pediatric cases. EMA testing demonstrates exceptional specificity, ranging from 98% to 100%, when performed by skilled laboratory personnel. As a result, EMA is widely recognized as the benchmark for CD-related serological testing [3].

While gluten is recognized as the main external factor in activating both innate and adaptive immune responses, it does not fully account for the complexity of CD pathogenesis [4,5,6]. Emerging hypotheses suggest that in genetically predisposed individuals, other factors, such as microbiota [7,8], the timing of gluten introduction in babies, mode of delivery at birth, breastfeeding, acute viral gastrointestinal infections, and deficiencies in micronutrients, may also play a role in the development of celiac disease [9,10,11].

CD frequently co-occurs with numerous immune-mediated conditions, including gastrointestinal disorders like ulcerative colitis, Crohn’s disease, and autoimmune liver disease, as well as immunoglobulin A (IgA) nephropathy, pernicious anemia, Addison’s disease, type 1 diabetes mellitus, and Sjögren’s syndrome, suggesting a shared underlying immunological mechanism [12,13].

There appears to be a bidirectional relationship between CD and autoimmune thyroid diseases (AITDs), with a significant rise in the incidence of AITD in those with CD and a correspondingly higher incidence of CD in those with AITD [13]. This connection may be caused by several mechanisms, such as common genetic factors, disruptions in the gut microbiota, and insufficiencies in essential micronutrients like vitamin D, which may contribute to a person’s heightened vulnerability to autoimmune diseases by compromising intestinal integrity and immune regulation [3,13].

Although research specifically addressing the role of vitamin D in CD remains limited, growing interest in this molecule—recognized for its potential in diverse biological functions—has led to an increasing number of studies exploring its involvement [14,15,16]. Recent studies show that vitamin D deficiency exists commonly among CD patients regardless of their intestinal mucosal damage severity. The research conducted by Infantino et al. showed that many CD patients had insufficient serum vitamin D levels, which indicates that vitamin D deficiency occurs independently of malabsorption [17]. A study conducted by Aydemir et al. revealed that vitamin D deficiency causes damage to intestinal epithelial integrity and affects bone metabolism in children with CD [18].

The immunological pathways through which vitamin D affects CD include multiple mechanisms. Vitamin D works as an anti-inflammatory agent through its ability to control T-regulatory cell development and activity while suppressing inflammatory cytokines. Vitamin D3 acts to block zonulin-mediated tight junction disruption which leads to preserved intestinal barrier integrity and possibly blocks gluten peptide movement into the lamina propria [19,20]. Vitamin D induces the production of antimicrobial peptides such as cathelicidin, which exhibit antibacterial, antifungal, and antiviral properties. These anti-infective properties could offer a mechanical link between the start of CD and vitamin D shortage [21].

Research now shows that specific vitamin D receptor (VDR) gene polymorphisms affect both the risk of developing CD and its disease severity. The meta-analysis by Lu et al. discovered that VDR genotypes might increase CD risk, which suggests vitamin D signaling pathways extend beyond basic deficiency mechanisms [22].

The aim of this study was to evaluate the association between suspected celiac disease and the coexistence of other autoimmune conditions, and to assess the relationship between autoimmune serology and serum vitamin D concentrations in the affected population.

## 2. Results

### 2.1. Study Group Characteristics

**Figure 1:**
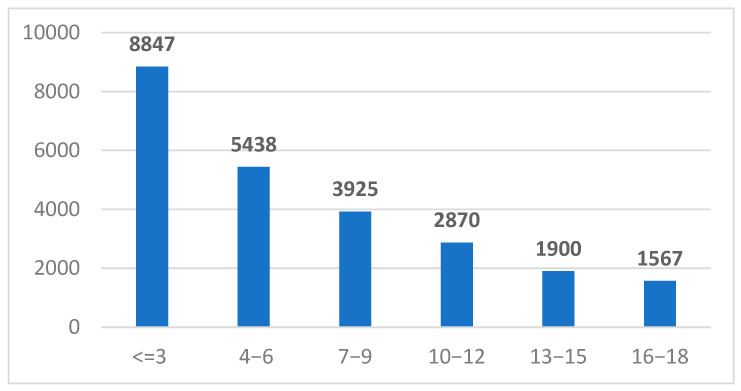
The distribution of children referred for anti-tTG antibody testing across defined pediatric age groups. The cohort was categorized into six age brackets: ≤3 years, 4–6 years, 7–9 years, 10–12 years, 13–15 years, and 16–18 years.

A total of 23,467 unique patients were included in this study, all of whom were registered in the Synevo laboratory information system between January 2019 and December 2024. These individuals, all residing in Romania, were referred for serological testing of IgA and IgG anti-tissue transglutaminase (anti-tTG) antibodies by healthcare providers across the country (Figure 1).

### 2.2. Characterization of Positive Anti-tTG (IgA and IgG) Population

**Figure 2:**
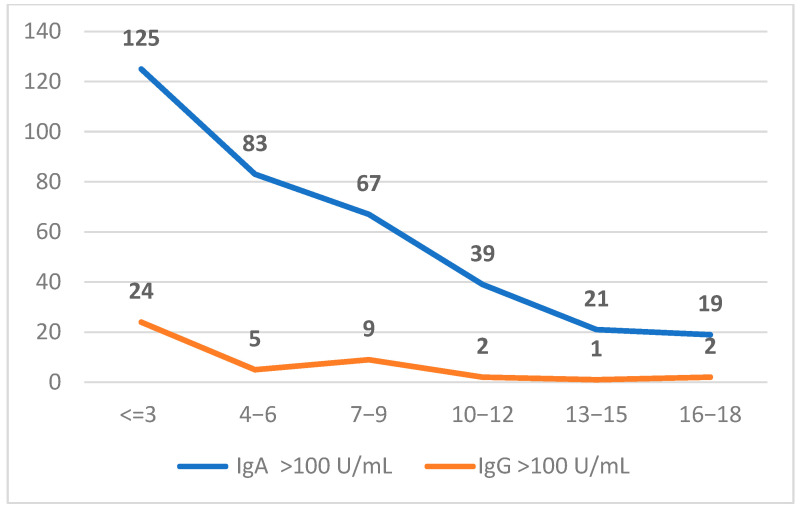
Distribution of anti-tTG IgG and IgA (>100 U/mL) results across pediatric age groups.

In accordance with the diagnostic recommendations outlined by the European Society for the Study of Coeliac Disease (ESsCD), we stratified our study cohort into three categories based on serological titers of anti-tTG antibodies: <10 U/mL, 10–100 U/mL, and >100 U/mL. Among the 23,467 unique patients included, 553 individuals exhibited IgA anti-tTG levels between 10–100 U/mL, while 342 exceeded 100 U/mL. In the IgG subgroup, 554 patients fell within the 10–100 U/mL range, and 43 had values >100 U/mL (Figure 2). Furthermore, patients were categorized into six age-based subgroups: <3 years, 3−6 years, 7−9 years, 10−12 years, 13−15 years, and 16−18 years. Notably, the highest proportion of positive anti-tTG results was observed in the <3 years age group, suggesting a greater likelihood of early serological manifestation of celiac disease in this cohort (Table 1).

### 2.3. Characterization of Positive Anti-Endomysium Antibody (IgA and IgG) Population

**Figure 3:**
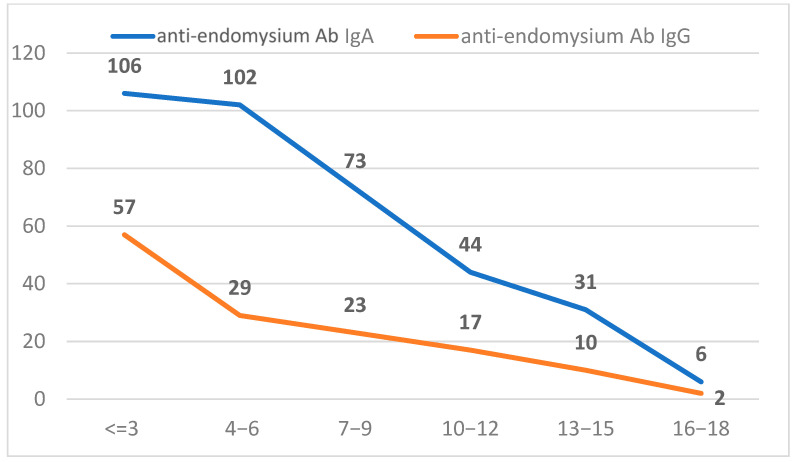
Distribution of anti-endomysium antibody IgG and IgA results across pediatric age groups.

We also analyzed a subset of the study population referred for anti-endomysium antibody (EMA) testing, comprising 3660 individuals for IgA EMA and 2898 for IgG EMA. Positivity was defined as a titer of ≥1:10. The overall positivity rate was 9.0% for IgA EMA and 4.6% for IgG EMA, indicating a higher seroprevalence of EMA IgA among the tested individuals (Figure 3).

### 2.4. Anti-TG IgA and Anti-Endomysium Antibodies IgA

Among the 342 children who tested positive for anti-tTG IgA antibodies, only 54.6% (n = 187) were subsequently referred for anti-endomysium antibody (EMA) IgA testing. Of those tested, 95% exhibited positive EMA IgA titers (≥1:10), indicating a strong concordance between elevated anti-TG IgA levels and EMA IgA positivity.

### 2.5. Anti-TG IgA and Anti-Gliadin Deamidated Antibodies (IgA and IgG)

We further examined the relationship between anti-tTG IgA and the presence of anti-gliadin deamidated (DGP) antibodies, both IgA and IgG. Among individuals with positive anti-tTG IgA results, 69 also tested positive for IgG DGP antibodies, while 24 were negative and 5 had equivocal results. Similarly, among those with positive anti-tTG IgA, 50 were also positive for IgA DGP, 41 were negative, and 10 had equivocal results. In total, 77 individuals showed positivity for anti-tTG IgA along with at least one positive DGP marker. It should be noted that the total number of cases presented in Table 2 represents the overall cohort of children which underwent testing for both IgA and IgG DGP antibodies (Table 2).

### 2.6. Anti-TG IgA and Genetic Predisposition for Celiac Disease

During the study period, 783 children (<18 years) were referred for genetic testing for celiac disease; however, only 88 of these individuals also underwent anti-tTG IgA testing. We further investigated the relationship between anti-tTG IgA positivity and genetic predisposition to celiac disease. Genetic testing for HLA-DQ2/DQ8 alleles, which are associated with increased susceptibility to celiac disease, was performed in 25.7% of anti-tTG IgA-positive individuals. Among those tested, 50% were positive for genetic markers linked to celiac disease, further supporting the role of HLA-associated genetic susceptibility in the serological diagnosis of the condition.

Among the 88 children tested, the majority (n = 79, 88.8%) carried HLA-DQ2 alleles. Specifically, 56 children (64%) carried the HLA-DQ2.5 haplotype (DQA1*05:01/DQB1*02:01), the most strongly associated genotype with celiac disease. An additional 16 children (18%) carried the DQA1*02:01/DQB1*02:02 variant, and 6 children (6.7%) had the DQA1*05:05/DQB1*02:02 genotype, both also classified under the broader DQ2 group.

Furthermore, 10 children (11.2%) were positive for HLA-DQ8 (DQA1*03/DQB1*03:02), another established susceptibility marker for celiac disease (Table 3).

### 2.7. Anti-tTG Antibodies and the Association with Other Autoimmune Antibodies

In our cohort of children, we examined the presence of several other autoantibodies to investigate potential associations with different autoimmune conditions. For thyroid autoimmunity, we assessed anti-thyroid peroxidase (anti-TPO) antibodies in 87 individuals, where 61 tested negative and 33 were positive.

Autoimmune diabetes markers were evaluated in children with celiac disease. Among 11 individuals tested for anti-glutamate decarboxylase (GAD) antibodies, 6 (54.5%) were positive and 5 (45.5%) negative, suggesting a potential overlap with autoimmune diabetes. Islet cell autoantibodies were assessed in 5 cases, with 2 (40%) positive and 3 (60%) negative. Anti-zinc transporter 8 (ZnT8) antibodies were measured in 2 children, showing 1 positive and 1 negative result. In addition, anti-thyroid peroxidase (anti-TPO) antibodies were detected in 11 of 29 individuals tested (37.9%). Overall, a subset of children with celiac disease exhibited additional autoimmune markers, with GAD and anti-TPO antibodies being most frequently detected, while islet cell and ZnT8 antibodies showed lower rates of positivity (Table 4).

### 2.8. Anti-TG IgA and 25(OH) Vitamin D Level

To explore the potential relationship between vitamin D status and children suspected of celiac disease, we analyzed anti-tTG IgA-positive samples (titers > 100 U/mL) stratified by age and serum 25(OH)D levels. Vitamin D concentrations were categorized as deficient (<20 ng/mL), insufficient (20–30 ng/mL), and sufficient (>30 ng/mL). In the youngest age group (≤3 years), 13 children were vitamin D deficient, 25 insufficient, and 42 had sufficient levels. Among children aged 4–6 years, 9 were deficient, 32 insufficient, and 40 sufficient. In the 7–9-year group, 13 had levels <20 ng/mL, 20 had levels between 20–30 ng/mL, and 28 exceeded 30 ng/mL. For the 10–12-year group, the distribution was 5, 16, and 9, respectively. Among adolescents aged 13–15, 6 were deficient, 8 insufficient, and 4 sufficient. In the 16–18-year group, only 1 individual was vitamin D deficient and 2 had insufficient levels; no subjects in this group had sufficient vitamin D concentrations (Table 5).

## 3. Discussion

This study provides a comprehensive laboratory-based assessment of serological, genetic, and biochemical profiles in a large cohort of Romanian pediatric patients suspected of having celiac disease. It integrates the analysis of anti-tTG, EMA, and DGP antibody profiles with HLA genotyping, autoimmune serological markers, and serum 25(OH)D quantification, offering an in-depth evaluation of diagnostic test utilization patterns in this clinical context.

The high concordance (95%) between anti-tTG IgA titers >100 U/mL and EMA positivity observed in our cohort underscores the strong diagnostic reliability of serological markers for CD. This finding aligns with the reported sensitivities and specificities of anti-tTG and EMA antibodies in pediatric populations, where EMA exhibits specificity rates exceeding 95% [23,24,25,26].

Moreover, the current study demonstrates a substantial correlation between anti-tTG IgA positivity and DGP antibody presence. Within our sample, 77 individuals tested positive for both markers, highlighting the complementary diagnostic role of DGP antibodies, especially in equivocal cases or in younger children where anti-tTG may occasionally yield false negatives. These observations align with current guidelines advocating DGP testing as a supplementary measure, especially in instances of IgA deficiency or variable serological profiles [27,28,29].

The genetic analysis in our study corroborates the recognized association of HLA-DQ2 and DQ8 genotypes with susceptibility to celiac disease. Among children tested, 88.8% carried HLA-DQ2 alleles, particularly the high-risk DQA1*05:01/DQB1*02:01 haplotype, while 11.2% were positive for HLA-DQ8. These findings are consistent with previous reports, indicating that approximately 90–95% of CD patients express HLA-DQ2, while the remainder express DQ8 [24,26,30].

It is crucial to recognize that although HLA testing possesses a high negative predictive value—effectively excluding celiac disease in the absence of DQ2 or DQ8—it does not provide adequate specificity for independent disease confirmation, given that these alleles are found in approximately 30–40% of the general European population [31]. Thus, genetic testing remains an essential but adjunctive tool.

Our analysis primarily concentrated on the correlation between vitamin D levels and CD seropositivity. A significant frequency of vitamin D insufficiency and inadequacy was noted among anti-tTG IgA-positive children, especially in younger cohorts. In the 7–9-year age group, 13 children were deficient in vitamin D, 20 were insufficient, and only 28 exhibited appropriate levels. This pattern aligns with growing research indicating that vitamin D deficiency is prevalent among newly diagnosed celiac disease patients, even prior to the initiation of a gluten-free diet [32,33,34]. Several mechanisms have been postulated, including malabsorption secondary to villous atrophy, chronic inflammation, and genetic polymorphisms affecting vitamin D metabolism [35].

Our findings underscore the necessity for systematic vitamin D screening at the time of celiac disease diagnosis, considering its implications for bone health, immunological function, and potential autoimmune comorbidities.

In accordance with the autoimmune characteristics of CD, we noted a significant incidence of additional autoantibodies among seropositive individuals. Of those examined, 37.9% tested positive for anti-TPO antibodies, indicating the presence of concurrent thyroid autoimmunity. Additionally, 6 out of 11 children screened were positive for anti-GAD antibodies, indicating a potential predisposition toward type 1 diabetes mellitus (T1DM) [36,37].

The correlation between celiac disease and other autoimmune disorders, especially autoimmune thyroiditis and type 1 diabetes mellitus, is well-established, characterized by common genetic predispositions—particularly HLA-DQ2/DQ8—and relevant environmental factors [38]. Notably, our findings emphasize the importance of proactive screening for additional autoimmune conditions in children diagnosed with CD, even in the absence of overt clinical symptoms.

Notably, the highest seropositivity rates for anti-tTG antibodies were recorded in children under three years old. The early serological manifestations in this cohort may indicate heightened genetic susceptibility or environmental factors, including premature gluten exposure or modified gut microbiota composition, all of which have been associated with the pathogenesis of celiac disease [39].

Vitamin D deficiency was notably more common in younger children, highlighting concerns that malabsorption and dietary deficiencies may have more significant impacts during crucial phases of growth and development.

Our findings highlight the necessity of a comprehensive approach to the diagnosis and therapy of CD, which includes serological testing, genetic risk assessment, vitamin D evaluation, and screening for concomitant autoimmune disorders. Given the correlation between positive serology and vitamin D inadequacy, we recommend the systematic assessment of serum 25(OH)D levels upon diagnosis, accompanied by prompt supplementation for those who are deficient or insufficient.

The robust correlation between anti-tTG IgA titers exceeding 100 U/mL and EMA positivity indicates that, in alignment with current ESPGHAN guidelines, intestinal biopsies may be judiciously excluded in certain high-titer instances, contingent upon the fulfillment of relevant clinical and genetic criteria [32].

This study presents several important limitations that should be acknowledged. First, the absence of clinical data—such as gastrointestinal symptoms, growth parameters, or family history of autoimmune disease—precludes confirmation of a celiac disease diagnosis and limits the interpretation of serological findings in a clinical context.

Second, this study assumed that all tested children were clinically suspected of having celiac disease, as testing was presumably initiated due to suggestive symptoms. However, without access to the clinical rationale for testing, there remains a degree of uncertainty regarding the pre-test probability and potential selection bias within the cohort.

Lastly, the observed associations between celiac-specific antibodies and other autoimmune markers (e.g., anti-TPO and anti-GAD antibodies) are based solely on serological data. In the absence of clinical manifestations or confirmatory diagnoses of comorbid autoimmune diseases, these findings represent potential immunological correlations rather than established comorbidities.

Future prospective studies should seek to fill these gaps and investigate therapies that may alleviate nutritional and immunological concerns in children with CD from the point of diagnosis onward.

## 4. Materials and Methods

### 4.1. Sample Population

We conducted a retrospective analysis of serological data from 23,467 unique patients referred to Synevo laboratories for IgA and IgG anti-transglutaminase antibodies between January 2019 and December 2024. Following the routing ordering process, all patients signed an informed consent form and blood samples were collected by specialized nurses. This study was approved by the Synevo laboratories internal ethical committee.

### 4.2. Laboratory Testing

For each patient, following blood collection, serum was separated and analyzed for celiac disease-specific serological markers, IgA and IgG anti-tissue transglutaminase (anti-tTG) antibodies. These tests were conducted in Synevo laboratories using commercially available assay kits, strictly adhering to the manufacturers’ protocols and internal standardized operational procedures.

In patients with detectable anti-tTG antibodies, clinicians also requested additional investigations which were performed to help CD diagnosis and also to assess broader autoimmune associations. These included testing for IgA and IgG anti-endomysium antibodies (EMAs), genetic susceptibility to celiac disease via HLA-DQ2/DQ8 typing, and autoantibodies associated with type 1 diabetes—such as anti-glutamate decarboxylase (GAD), anti-pancreatic islet cell antibodies, and anti-zinc transporter 8 (ZnT8) antibodies. Evaluation of thyroid autoimmunity was also conducted through measurement of anti-thyroid peroxidase (anti-TPO) antibodies. In addition, serum 25(OH) vitamin D levels were assessed to explore potential correlations between celiac disease, coexisting autoimmune conditions, and vitamin D status.

### 4.3. Anti-Tissue Transglutaminase IgA and IgG

The anti-tissue transglutaminase IgA and IgG test is an ELISA system used for the quantitative determination of IgA and IgG class autoantibodies against tissue transglutaminase (tTG) in human serum. The method is based on an indirect enzyme-linked immune reaction. In interpreting the results, values below 10 U/mL were classified as negative, whereas values of 10 U/mL or higher were considered positive.

### 4.4. Anti-Endomysium Antibodies (EMAs)

The EMA IgA and IgG tests are indirect immunofluorescence assays (IIFAs) using monkey esophagus sections (Aesku Diagnostics kit). If the patient’s serum contained specific antibodies, they would bind to the antigens on the slide during the first incubation step. After a washing step to remove unbound components, the bound antibodies would be detected using a fluorescein-labeled anti-human immunoglobulin, applied during a second incubation. A positive result was recorded if the connective tissue surrounding the muscle cells was brightly fluorescent, forming a honeycomb pattern.

### 4.5. HLA-DQ2/DQ8

Testing for celiac disease susceptibility genes was performed using polymerase chain reaction (PCR) with allele-specific primers, targeting the identification of HLA-DQ2 and HLA-DQ8 alleles. DNA was isolated from nucleated cells by digestion with proteinase K, and HLA class II low-resolution genotyping was performed.

### 4.6. Anti-TPO

The immunological test for the quantitative determination of anti-thyroid peroxidase (anti-TPO) antibodies in human serum was performed using Elecsys kits based on the electrochemiluminescence immunoassay (ECLIA) for Cobas e801 immunoassay analyzers (Roche Diagnostics GmbH, Mannheim, Germany). The Elecsys Anti-TPO reagent was used for testing, with a measurement range of 9–600 IU/mL. Results below the detection limit were reported as <9 IU/mL, while those exceeding the upper measurement range were reported as >600 IU/mL.

### 4.7. Anti-GAD

The anti-GAD test used is an ELISA-based assay designed for the quantitative measurement of autoantibodies against glutamate decarboxylase (GAD) in human serum. According to the manufacturer, EUROIMMUN (Lübeck, German), the results below 10 IU/mL are interpreted as negative, while values equal to or exceeding 10 IU/mL are considered positive.

### 4.8. Zinc Transporter 8 Antibodies

Antibodies against ZnT8 are primarily directed against the C-terminal domain of the zinc transporter 8 (ZnT8) protein. The ELISA test for detecting anti-ZnT8 autoantibodies is based on the bridging principle, which utilizes the bivalent nature of ZnT8 antibodies, allowing them to bind with one arm to ZnT8 coated on the plate well and with the other arm to biotin-labeled ZnT8 in solution. The reference value is <15.0 U/mL. Values ≥ 15 IU/mL may support the diagnosis of type 1 diabetes or guide therapeutic decisions for patients with diabetes.

### 4.9. 25 OH-Vitamin D

The 25 OH-Vitamin D test is a binding assay used for the quantitative in vitro determination of total 25-hydroxyvitamin D in human serum. The electrochemiluminescence binding assay is designed for use on Roche Cobas e801 immunology analyzers. The Elecsys Vitamin D total III test utilizes a vitamin D binding protein labeled with a ruthenium complex as a capture protein for the binding of 25-hydroxyvitamin D3 and 25-hydroxyvitamin D2, using the competition principle. It was noted that cross-reactivity with 24,25-dihydroxyvitamin D is inhibited by a specific monoclonal antibody. Regarding the measurement range, it was mentioned that it spans from 3.00 to 120 ng/mL. Values exceeding this range are reported as >120 ng/mL or up to 240 ng/mL for samples diluted.

### 4.10. Ethics and Integrity Statement

The authors affirm that the research presented in this manuscript complies with the journal’s ethics policies. Informed consent was obtained from all participants involved in this study, and steps were taken to protect their privacy and confidentiality. This study was conducted in accordance with the principles of the Declaration of Helsinki.

The authors declare that the research methodology, data collection, analysis, and interpretation presented in this manuscript adhere to the highest standards of scientific integrity. Any potential conflicts of interest that could influence the research findings have been transparently disclosed.

## 5. Conclusions

This study demonstrates the necessity of using serological, genetic, and biochemical profiling methods for diagnosing and treating celiac disease in Romanian children. It emphasizes that combining anti-tTG and EMA antibodies with DGP testing enhances diagnostic precision. Given the higher presence of anti-TPO and anti-GAD antibodies, the results also highlight the need of screening for related autoimmune diseases. The rather high frequency of vitamin D deficiency among seropositive patients also underscores the need for routine monitoring and timely supplementation following diagnosis.

Considering the accumulating evidence connecting vitamin D deficiency to the severity of CD and the risk of other autoimmune disorders, additional longitudinal studies are necessary to elucidate causality and to ascertain whether early restoration of vitamin D levels can influence disease progression. Moreover, investigating the role of microbiome composition, genetic modifiers beyond HLA-DQ2/DQ8, and environmental exposures in the Romanian pediatric population may yield important insights into CD pathogenesis and prevention strategies.

Our research demonstrates that children with celiac disease need a multidisciplinary approach for diagnosis and management which includes clinical evaluation, serological testing, genetic assessment, nutritional management, and immunological assessment to enhance treatment quality.

## Figures and Tables

**Table 1:** The table presents the distribution of anti-tTG IgA and IgG results across pediatric age groups, categorized according to EFSGAN guidelines into three serological ranges: <10 U/mL (negative), 10–100 U/mL (moderate/equivocal), and >100 U/mL (strongly positive).

Age Groups (Years)	<10 U/mL		10–100 U/mL		>100 U/mL	
	**IgG**	**IgA**	**IgG**	**IgA**	**IgG**	**IgA**
<=3	8635	8651	122	235	125	24
4−6	5312	5232	176	153	83	5
7−9	3842	3794	119	89	67	9
10−12	2821	2766	86	56	39	2
13−15	1881	1847	55	22	21	1
16−18	1547	1523	41	22	19	2
**Total**	**23,027**	**22,882**	**553**	**554**	**342**	**43**

**Table 2:** Distribution of anti-DGP among children with suspected celiac disease.

Anti-Gliadin Deamidated Antibodies	Equivocal	Negative	Positive
IgG	5	24	69
IgA	10	41	50
**Total**	**14**	**44**	**77**

**Table 3:** The distribution of HLA types among the children tested shows that the majority carried celiac disease-associated alleles, with 78 (89.7%) testing positive for HLA-DQ2 variants and 10 (11.5%) for HLA-DQ8.

HLA Type	Number of Children
HLA-DQ2 (DQA1*02:01/DQB1*02:02)	16
HLA-DQ2 (DQA1*05:01/DQB1*02:01)	56
HLA-DQ2 (DQA1*05:05/DQB1*02:02)	6
HLA-DQ8 (DQA1*03/DQB1*03:02)	10

The * is part of the way of reporting HLA haplotypes.

**Table 4:** Frequency of additional autoimmune markers in children diagnosed with celiac disease.

	Negative	Positive
Anti-TPO (anti-thyroid peroxidase)	61	33
Anti-glutamate decarboxylase (GAD) antibodies	5	6
Anti-pancreatic islet cell antibodies	3	2
Anti-transporter 8 zinc antibodies	1	1

**Table 5:** Distribution of positive sera (>100 U/mL) for anti-tTG according to age groups and level of 25(OH) vitamin D.

Age Groups (Years)	<20 ng/mL	20–30 ng/mL	>30 ng/mL
<=3	13	25	42
4–6	9	32	40
7–9	13	20	28
10–12	5	16	9
13–15	6	8	4
16–18	1	2	0
**Total**	**47**	**103**	**123**

## Data Availability

The original contributions presented in this study are included in the article; further inquiries can be directed to the corresponding author.
